# Management of cancer-related fatigue in Taiwan: an evidence-based consensus for screening, assessment and treatment

**DOI:** 10.1093/jjco/hyac164

**Published:** 2022-11-09

**Authors:** Kun-Ming Rau, Shiow-Ching Shun, Shih-Hsin Hung, Hsiu-Ling Chou, Ching-Liang Ho, Ta-Chung Chao, Chun-Yu Liu, Ching-Ting Lien, Ming-Ying Hong, Ching-Jung Wu, Li-Yun Tsai, Sui-Whi Jane, Ruey-Kuen Hsieh

**Affiliations:** Department of Hematology Oncology, E-Da Cancer Hospital, Kaohsiung, Taiwan; School of Medicine, College of Medicine, I-Shou University, Kaohsiung, Taiwan; College of Nursing, Institute of Clinical Nursing, National Yang Ming Chiao Tung University, Taipei, Taiwan; Department of Nursing, Taipei Veterans General Hospital, Taipei, Taiwan; Department of Nursing, Asia Eastern University of Science and Technology, New Taipei City, Taiwan; Department of Nursing, Far Eastern Memorial Hospital, New Taipei City, Taiwan; School of Nursing, National Yang Ming Chiao Tung University, Taipei, Taiwan; Division of Hematology and Oncology, Tri-Service General Hospital, Taipei, Taiwan; Division of Oncology, National Defense Medical Center, Taipei, Taiwan; Department of Oncology and Comprehensive Breast Health Center, Taipei Veterans General Hospital, Taipei, Taiwan; Faculty of Medicine, School of Medicine, National Yang Ming Chiao Tung University, Taipei, Taiwan; Division of Transfusion Medicine, Department of Medicine and Comprehensive Breast Health Center, Taipei Veterans General Hospital, Taipei, Taiwan; Division of Medical Oncology, Department of Oncology, Taipei Veterans General Hospital, Taipei, Taiwan; School of Medicine, National Yang Ming Chiao Tung University, Taipei, Taiwan; Department of Nursing, MacKay Memorial Hospital, Taipei, Taiwan; Department of Nursing, National Taiwan University Hospital, Taipei, Taiwan; Department of Radiation Oncology, Cathay General Hospital, Taipei, Taiwan; Department of Radiation Oncology, National Defense Medical Center, Taipei, Taiwan; Department of Biomedical Engineering, I-Shou University, Kaohsiung, Taiwan; College of Nursing, Central Taiwan University of Science and Technology, Taichung, Taiwan; Division of Hematology-Oncology, Department of Internal Medicine, Chang Gung Memorial Hospital, Taoyuan, Taiwan; Graduate Institute of Nursing, Chang Gung University of Science and Technology, Taoyuan, Taiwan; Department of Hematology and Oncology, MacKay Memorial Hospital, Taipei, Taiwan

**Keywords:** cancer-related fatigue, oncology, oncology nursing, palliative care, quality of life

## Abstract

**Background:**

Cancer-related fatigue is one of the most common and persistent issues experienced by cancer patients. Cancer-related fatigue is a distinct form of fatigue that is subjective, long-lasting and unalleviated by rest or sleep. Studies have shown that almost all cancer patients experience severe fatigue that disrupts the quality of life and physical function, but cancer-related fatigue remains under-addressed in clinical care, and only about half of all patients receive treatment.

**Methods:**

To increase the awareness of cancer-related fatigue and improve current management, the Taiwan Society of Cancer Palliative Medicine and the Taiwan Oncology Nursing Society convened a consensus committee to develop recommendations for the screening, assessment and treatment of cancer-related fatigue.

**Results:**

Thirteen consensus recommendations were subsequently developed based on the best available evidence and the clinical experience of committee members.

**Conclusions:**

These recommendations are expected to facilitate the standardization of cancer-related fatigue management across Taiwan and may also serve as a reference for other clinicians.

## Teaser

Cancer-related fatigue should be evaluated regularly, and non-pharmacological and pharmacological interventions are critically needed.

## Introduction

Since 1982, cancer has been the leading cause of death in Taiwan (1). Close to 110 000 patients are newly diagnosed with the disease every year (2). It is known that the symptoms and treatments of cancer can adversely impact physical and psychological function in cancer patients. Of the discomforts experienced, cancer-related fatigue (CRF) has consistently been the most frequent and persistent issue reported by patients (3–5). CRF is a distinct and subjective form of fatigue that is typically long-lasting and unalleviated by rest or sleep (4). Studies have shown that 59–100% of cancer patients experience debilitating fatigue that drains physical energy, induces loss of interest in daily activities, reduces function, increases stress, causes sleep disturbances, and generally contributes to poor quality of life (QoL) (4,6,7). The causes of CRF include underlying cancer status, side effects from treatments and psychological problems. In addition, CRF is a common and severe side effect of immunotherapy (8). However, CRF remains under-addressed in the clinical setting. A recent large-scale study conducted across 23 hospitals in northern, central and southern Taiwan, involving 1207 cancer patients, found that 92% of patients had experienced fatigue-related issues, with about 25% experiencing moderate to severe fatigue (9). In addition, a meta-analysis of 129 studies published between 1993 and 2020 from across Europe (72 studies), North America (33 studies), Asia (18 studies, including 3 studies from Japan and 2 from Taiwan) and other areas (6 studies) showed overall CRF prevalence to be 49% among cancer patients, rising to 62% in patients receiving anticancer treatment (10). These results show that CRF is a global issue that remains inadequately addressed (10). This is likely because patients are often reticent about mentioning their fatigue issues to healthcare personnel; for instance, the large-scale study conducted in Taiwan found that although 83.5% of patients took measures to relieve their fatigue, just 56.2% of patients voluntarily reported their fatigue issues to healthcare personnel, and only 54.8% of healthcare personnel provided fatigue-relieving measures (9). This study further showed that fatigue was ranked as having a greater disruptive impact than sleep disturbances, pain, loss of appetite and depression, but patients were often unwilling or unable to describe their fatigue to healthcare personnel (9). Other research has shown that patients often choose to bear their fatigue in silence, for fear of distracting or increasing the workload of doctors and nursing staff (11). For example, a study of 200 American cancer patients found that 66% had never spoken with their attending physician about their fatigue, primarily because physicians did not proactively offer fatigue-relieving interventions (47%), patients were unaware that fatigue treatments were available (43%), patients did not wish to treat fatigue using pharmacologic methods (40%), or patients simply did not want to complain about their fatigue to healthcare personnel (28%) (12).

The lack of a clinical management consensus for CRF has meant that related issues are often overlooked in clinical practice by healthcare personnel and patients. In light of this, the Taiwan Society of Cancer Palliative Medicine (TSCPM) and the Taiwan Oncology Nursing Society (TONS) decided to convene a consensus committee to develop clinical recommendations for the screening, assessment and treatment of CRF, based on the best available evidence and the clinical experience of committee members.

## Materials and methods

### Composition of the consensus committee

The consensus committee comprised 38 members, including 31 clinicians and 7 nursing experts, all of which had relevant experience in the clinical management, education and/or research of CRF. Committee members were subsequently assigned to one of two working subgroups as based on their expertise, one for the assessment of pharmacological treatments, and the other for the assessment of non-pharmacological treatments.

### Literature review and consensus development

In the first stage of consensus development, the consensus committee determined the scope of the consensus to include recommendations for the screening, assessment and treatment of CRF in the clinical setting for Taiwan. Committee members then worked together to conduct a review of the literature published between January 2012 and July 2017, using the PubMed, MEDLINE, PsycINFO and CINAHL (Cumulative Index to Nursing and Allied Health Literature) databases. The keywords, ‘cancer’, ‘fatigue’, ‘treatment’ or ‘herbal’ were used to identify literature pertaining to pharmacological treatments, for which only randomized controlled trials (RCTs) were included; while the keywords, ‘cancer’, ‘fatigue’, ‘exercise’, ‘physical activity’, ‘psychological’, ‘nutrition’, ‘sleep’ or ‘alternative therapy’ were used to identify literature regarding non-pharmacological treatments, for which all clinical studies were included. A total of 7547 individual papers were identified from the initial literature review, and after initial review and discussion, the consensus committee elected to include all RCTs of pharmacological treatments and only well-designed RCTs, systematic reviews and meta-analyses of non-pharmacological treatments in the development of the consensus. Subsequently, 60 studies were included. Three committee meetings were held from August to October 2017, and the finalized Chinese version of the consensus was presented at the TSCPM annual meeting in November 2017, where public comments and feedback from external experts and patient groups were sought and received. Following revision based on this feedback, the finalized Chinese version of the consensus was presented at the 2018 Taiwan Joint Cancer Conference. At that time, it was suggested that an English version of the consensus should be developed and published in an international journal.

In the second stage of consensus development, an English manuscript was developed from the Chinese consensus. Five consensus committee meetings were held over 2019–20 to review the literature published between August 2017 and July 2019, and revise the recommendations to reflect the latest evidence. The English version of the consensus was approved by the committee in February 2020. Following presentation of this English consensus at the annual meetings of oncology-related societies in Taiwan, comments and feedback were collected from non-participating experts and patient groups. These comments and an additional review of the literature from August 2019 to October 2021 were used to revise and finalize the English version of the consensus. During this stage, an additional 23 studies were eventually included.

### Grading of recommendations

Based on the initial literature review, committee members proceeded to develop recommendations for the screening, assessment, and treatment of CRF, and 13 recommendations were eventually proposed to the consensus committee for approval. Several possible recommendations were discussed but were ultimately not proposed to the committee because of limited evidence. The threshold required for inclusion of recommendations was unanimous consent, and if objections were raised during the initial round of voting, the wording of the recommendation was revised and another round of voting was conducted. All recommendations and accompanying statements were subjected to word-by-word review and revision in the consensus committee, until complete consensus was reached, with all 13 proposed recommendations accepted by the consensus committee within two rounds of voting. The level of evidence and grade of recommendation for each consensus recommendation was subsequently determined using the US National Guideline Clearinghouse standards (Table 1) (13).

**Table 1:** Levels of evidence and grades of recommendations used by the US National Guideline Clearinghouse

Levels of evidence	
IA	Evidence from meta-analysis of randomized controlled trials
IB	Evidence from at least one randomized controlled trial
IIA	Evidence from at least one controlled study without randomization
IIB	Evidence from at least one other type of quasi-experimental study
III	Evidence from non-experimental descriptive studies, such as comparative studies, correlation studies, and case–control studies
IV	Evidence from expert committee reports or opinions or clinical experience of respected authorities, or both
Grades of recommendation	
A	Directly based on Level I evidence
B	Directly based on Level II evidence or extrapolated recommendations from Level I evidence
C	Directly based on Level III evidence or extrapolated recommendations from Level I or II evidence
D	Directly based on Level IV evidence or extrapolated recommendations from Level I, II, or III evidence

## Results

CRF is a distinct form of fatigue that is defined in the US National Comprehensive Cancer Network (NCCN) Clinical Practice Guidelines in Oncology for Cancer-Related Fatigue, version 2.2022, as being ‘a distressing, persistent, subjective sense of physical, emotional, and/or cognitive tiredness or exhaustion related to cancer or cancer treatment that is not proportional to recent activity and interferes with usual functioning’ (14). Based on this definition, the consensus committee conducted a literature review of studies and developed 13 consensus recommendations regarding the definition, assessment and management of CRF (Table 2). These recommendations and their underlying evidence are discussed in the following sections.

**Table 2:** Overview of consensus recommendations

Definition of cancer-related fatigue **Recommendation 1:** Cancer-related fatigue is a form of severe fatigue associated with cancer or cancer treatment. Symptoms are disproportionate to levels of physical activity, and are typically subjective, long-lasting, distressing for patients and disruptive to daily life. (Level of evidence: IV; Grade of recommendation: D.) Screening and assessment **Recommendation 2:** In clinical practice, cancer-related fatigue should be regularly and continuously monitored, managed/treated, prevented and reassessed. Assessment of cancer-related fatigue should be conducted for cancer patients during their first visit, for hospitalized patients every day, and for outpatients at each return visit. Patients may assess and record their own fatigue levels using a fatigue diary. (Level of evidence: IV; Grade of recommendation: D.) **Recommendation 3:** Considering that fatigue is a prevalent but often overlooked issue in cancer patients, upon diagnosis of cancer and at return visits thereafter, all patients should undergo a simple fatigue assessment. Patients should be asked if they feel fatigued, after which they can express their fatigue levels using the Visual Analog Scale or Numerical Rating Scale. Mild fatigue can be addressed through non-pharmacological management, but for moderate to severe fatigue with a Numerical Rating Scale score ≥ 4, special attention is required, and a comprehensive fatigue assessment is needed, followed by the development of a treatment strategy in which the addition of pharmacological therapies should be considered. After the start of treatment, the frequency of assessment is at the discretion of the attending physician. (Level of evidence: IV; Grade of recommendation: D.) Non-pharmacological treatment **Recommendation 4:** Cancer patients should receive general care upon initial diagnosis, such as patient education and energy conservation and activity management measures, to enhance their awareness of fatigue and assessment capabilities; in addition, patients should be educated in the recording and review of their fatigue severity, in order to help them find their optimal pace of life and seek assistance from healthcare professionals if needed. (Level of evidence: IB; Grade of recommendation: B.) **Recommendation 5:** Maintaining regular exercise during and after cancer treatment is helpful for improving cancer-related fatigue in all types of cancer patients. When designing exercise programs, it is recommended to increase exercise intensity gradually, in increments of less than 60–75% of heart rate for the previous session; moreover, all exercise should include a 5-min warm-up before and a 5-min cool-down afterward, to ensure safe execution of the exercise program. Patient limitations should be noted during exercise, and patients should be referred to the relevant professional personnel when necessary. (Level of evidence: IA; Grade of recommendation: A.) **Recommendation 6:** Psychosocial interventions alone or combined with exercise are effective for reducing cancer-related fatigue. For patients who have completed cancer treatment, face-to-face group cognitive-behavioral therapy has been shown to be the most effective. (Level of evidence: IA; Grade of recommendation: A.) **Recommendation 7:** Current clinical evidence suggests that sleep hygiene measures should be combined with other non-pharmacological interventions, to have a better chance of improving cancer-related fatigue. (Level of evidence: IB; Grade of recommendation: B.) **Recommendation 8:** Clinical evidence shows that high-fiber and low-fat diets rich in fruits, vegetables, whole-grains and foods containing large amounts of omega-3 polyunsaturated fatty acids can help to improve cancer-related fatigue, and consultation or referral to a dietician for dietary planning is recommended. (Level of evidence: IB; Grade of recommendation: B.) **Recommendation 9:** Preliminary clinical evidence shows that acupuncture, acupressure and massage can help to improve cancer-related fatigue; however, the attending physician should be informed before undergoing any complementary therapies, and consultation or referral to the relevant professional personnel should be conducted if necessary. (Level of evidence: IB; Grade of recommendation: B.) Pharmacological treatment **Recommendation 10:** Clinical evidence shows that methylphenidate might be effective for patients with severe fatigue or advanced disease; however, dosage, treatment duration, risk of abuse and individual disease characteristics should be carefully considered prior to initiating treatment, to ensure that associated risks and benefits are fully evaluated. (Level of evidence: IA; Grade of recommendation: A.) **Recommendation 11:** Clinical evidence indicates that steroids are able to improve fatigue and quality of life for cancer patients. However, the long-term use of steroids carries safety risks, and thus treatment is only recommended for cancer patients with terminal disease, combined with fatigue, anorexia or pain caused by brain or bone metastasis. (Level of evidence: IB; Grade of recommendation: B.) **Recommendation 12:** Locoregional clinical trials indicate that injection of *Astragalus* polysaccharides can improve moderate to severe cancer-related fatigue. (Level of evidence: IA; Grade of recommendation: A.) Traditional herbal medicine **Recommendation 13:** Clinical trials indicate that ginseng can improve cancer-related fatigue; however, because traditional Chinese medicine may be influenced by factors such as preparation processes of raw materials, it is recommended that the patient’s healthcare team should be consulted before initiating treatment. (Level of evidence: IB; Grade of recommendation: B.)

Note: Levels of evidence and grades of recommendation were derived from the US National Guideline Clearinghouse (10): https://hsl.lib.umn.edu/biomed/help/levels-evidence-and-grades-recommendations.

### Definition of cancer-related fatigue

The consensus committee established a clear definition of CRF at the outset, based on the latest version of the NCCN CRF guidelines (14) and the 10th revision of the International Statistical Classification of Diseases and Related Health Problems (ICD-10) (11). Recommendation 1 presents this definition and includes the key features of CRF. This is expected to assist clinicians and nursing staff in the detection and evaluation of CRF.

### Screening and assessment

Several clinical surveys have shown that CRF is prevalent but often under-detected in cancer patients (4,6,7,9). Therefore, the consensus committee follows the latest NCCN CRF guidelines (14) in recommending that CRF assessments should be conducted for all cancer patients during their first visit, with outpatients to receive assessments at each return visit, while hospitalized patients should be assessed daily. This is to ensure that CRF can be detected and managed in a timely fashion, in order to minimize potential disruptions to cancer treatment and deteriorations in QoL. Recommendation 2 describes the optimal screening process for CRF, with an emphasis on regular and continuous monitoring. Patient self-evaluation measures such as keeping a fatigue diary are also encouraged. To ensure timely assessment and management of CRF, the consensus committee recommends that a Visual Analog Scale (VAS) or Numerical Rating Scale (NRS) be used for initial assessment to determine the overall level of fatigue experienced by the patient, followed as needed by a comprehensive fatigue assessment that includes aspects such as the patient’s current diagnosis and staging, treatment regimen, review of bodily systems, fatigue review (onset, patterns, duration, changes over time, triggering/relieving factors and impact on daily life), social support situation and evaluation of possible causes (treatment, pain, anemia, emotional discomfort, reduced fitness, sleep issues, nutritional issues or other comorbidities) (14). Considering that CRF is often under-assessed by physicians (9), the consensus committee opted to use the quick and simple VAS/NRS for initial assessment, as physicians can use the very short time available during outpatient or bedside visits to rapidly assess the pervasive issue of fatigue in cancer patients, without having to get into complicated details regarding frequency, severity, interference, or causes. As CRF assessment becomes more embedded into routine practice, future revisions to this approach may be considered.

**Figure 1:**
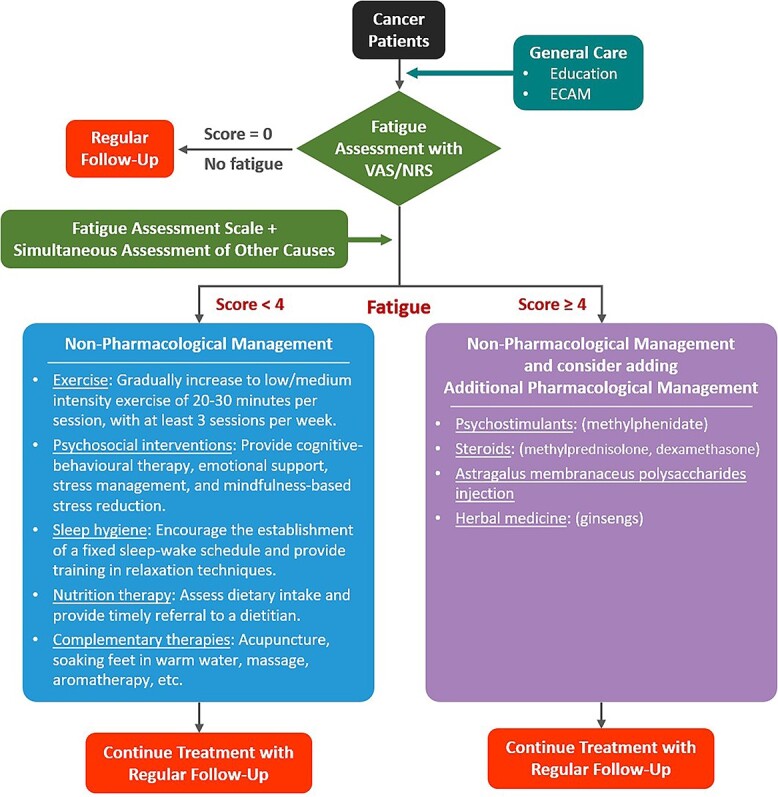
Algorithm for the standardized assessment and management of cancer-related fatigue. ECAM, energy conservation and activity management; VAS, Visual Analog Scale; NRS, Numerical Rating Scale.

Importantly, as a previous national survey of 1207 cancer patients from 20 hospitals found that 78.7% of patients with a VAS/NRS (0–10 points in ascending severity) fatigue score > 3.5 points conformed with a diagnosis of CRF based on the ICD-10 criteria (9), the consensus committee decided that for patients with VAS/NRS fatigue scores ≥ 4 points, the threshold for moderate to severe fatigue as defined by the NCCN CRF guidelines (14) and other reviews (15,16), healthcare providers should offer in-depth assessment and consider combining non-pharmacological and pharmacological treatments. The committee has developed an algorithm to standardize the assessment and management of CRF in a clinical setting (Fig. 1), and Recommendation 3 describes the main principles of this algorithm.

### Non-pharmacological treatment

Non-pharmacological treatment plays an important role in the management of CRF, as pharmacological treatment is still quite limited; moreover, there is a growing body of evidence that indicates non-pharmacological treatment, when undertaken by trained health professionals in a standardized way, can help to alleviate the severity and disruptive impact of CRF (4,14,16). The recommendations in this section therefore cover the current range of non-pharmacological measures available for the management of CRF, including patient education, exercise, psychosocial interventions, sleep, nutrition and complementary therapies.

#### Patient education

A recent Cochrane review has shown that patient education can provide mild relief for CRF and reduce disruptions to daily life, but only in cancer patients that have not reached an advanced stage; moreover, there is insufficient evidence to support any specific educational intervention (17). However, based on clinical experience, the consensus committee believes that it is very important to educate cancer patients that CRF does not indicate their cancer is worsening, but may actually be a side effect of treatment. This is to ensure that patients do not become afraid of acknowledging or describing their fatigue symptoms to healthcare personnel. In addition, evidence from RCTs suggests that educating patients to conduct daily tracking and recording of their own fatigue levels, as well as the impact of fatigue on physical function, can help to alleviate fatigue (18,19).

#### Energy conservation and activity management

Patients can also benefit from learning about energy conservation and activity management (ECAM) measures, including setting priorities, delegating, planning, acting during times of peak energy, pacing, and rest (20–22), as these have been shown in RCTs to reduce fatigue significantly (23,24). Based on expert opinion, the consensus committee suggests that ECAM measures should be taught over 3 weeks, with patients receiving face-to-face instruction during the first chemotherapy session, followed by two telephone consultations to be conducted over the next 2 weeks. Nursing staff will conduct telephone consultations according to a standardized structure. If fatigue symptoms persist, assistance from other specialists or healthcare professionals (e.g. dieticians) should be sought (18). Recommendation 4 presents best practices for general care and patient education in cancer patients diagnosed with CRF.

#### Exercise

Of all non-pharmacological management measures for CRF, exercise has the strongest evidence (25–30) and has been shown to be effective in reducing fatigue at all stages of cancer management and for multiple types of cancer (31–36). Based on the current evidence, the consensus committee recommends the design of an individualized exercise program, starting from low-intensity exercise according to the patient’s condition, and then gradually adjusting intensity (aiming to reach a 60–75% heart rate increase over the previous session) until low- or medium-intensity exercise of 20–30 min per session, with at least three sessions per week, can be achieved (14,26,37). The content of the exercise should be based on personal preference, but all exercise programs should include a 5-min warm-up before and a 5-min cool-down afterward, to ensure patient safety (37,38). In addition, although there is strong evidence that exercise can help to alleviate CRF (39–46), patients with bone metastases, abnormal blood cell counts, anemia, fever, infection or other serious symptoms or comorbidities should avoid exercise, and in case of doubt, a relevant professional should be engaged to assist in the development of an appropriate exercise program (14).

#### Psychosocial interventions

Current evidence shows that psychosocial interventions alone or combined with exercise can be effective in reducing CRF (47–50), with RCTs indicating that face-to-face group cognitive-behavioral therapy (CBT) is most effective in reducing fatigue for patients that have completed cancer treatment (51,52). The consensus committee further recommends that all healthcare personnel responsible for conducting psychosocial interventions or CBT should have the ability to evaluate CRF and psychological stress in patients, educate patients regarding CRF and assess whether referral to a therapist would be necessary. Recommendation 6 presents the evidence regarding psychosocial interventions for the management of CRF.

#### Sleep hygiene

Sleep hygiene aims to improve sleep quality through the development of good sleeping habits. Although existing clinical evidence showed that improving sleep does not directly decrease the intensity of CRF, poor sleep quality can worsen fatigue (53,54). In addition, sleep hygiene can be combined with other non-pharmacological interventions (e.g. CBT) to have maximum effect (55–57). Behavioral, cognitive and educational intervention strategies can be effective in improving sleep quality, but it should be noted that patients with hallucinations, delusions, severe mental disorders, depression or severe cognitive impairment are not suitable for cognitive therapy. In addition, the consensus committee recommends that healthcare personnel responsible for sleep education should be able to assist patients in developing good sleep hygiene as well as train patients in relaxation techniques to improve sleep quality. Recommendation 7 further emphasizes that sleep hygiene measures should be combined with other non-pharmacological interventions to have a better effect on CRF.

#### Nutrition

The dietary intake of cancer patients is often affected by the disease itself, as well as related treatment, and therefore the nutritional status of patients should be regularly assessed and monitored to avoid malnutrition and subsequent disruption of treatment. Individual nutrition consultation or supplementation should also be conducted in a timely fashion, and referral to a dietician may be helpful. Current evidence from RCTs and large observational trials indicates that high-fiber and low-fat diets that are rich in fruits, vegetables, whole-grains and omega-3 polyunsaturated fatty acids can help to alleviate CRF (58–63), while limited evidence suggests that the addition of specific nutritional supplements may be beneficial (64–66). However, food that is too hard and rough may increase the risk of tumor bleeding, so high-fiber diets are not recommended for patients with colorectal cancer, gastric cancer, or esophageal varices caused by liver cancer with liver cirrhosis. The consensus committee further recommends that healthcare personnel responsible for nutrition assessment and management must be able to assess nutritional status and record dietary intake and provide nutritional consultation to guide patients on how to consume a balanced diet (taking the patient’s dietary preferences and views into account). Recommendation 8 notes the dietary patterns that can help to improve CRF and emphasizes the importance of including a dietician in dietary planning for CRF management.

#### Complementary therapies

Complementary therapies beyond the scope of conventional medicine are increasingly being sought by cancer patients, and many cancer centers around the world have begun to incorporate complementary therapies into standard cancer care as well. The consensus committee reviewed the evidence regarding the effects of complementary therapies on CRF. As of now, larger RCTs have mainly investigated the effects of acupuncture and massage on CRF, and the remaining studies are mostly small and lack rigorous controls. A meta-analysis comprising seven trials has shown that acupuncture can significantly improve CRF as compared with acupressure or sham acupuncture (67), and limited evidence supports the benefits of acupressure over sham acupuncture as well (68). In addition, there are two RCTs that suggest the use of Swedish massage can improve fatigue to a greater degree than that observed in controls (69,70). Based on this evidence, the consensus committee believes that complementary therapies can be useful and recommends that healthcare professionals should respect patients’ decisions regarding complementary therapies and actively engage with patients regarding the selection and monitoring of such therapies, as long as formal treatment and safety are not compromised. However, the consensus committee recommends that patients should inform their attending physicians and receive approval prior to initiating any therapy, and a detailed medical history, previous experience with any complementary therapy and a fatigue assessment should be obtained from the patient prior to starting any therapy, in order to individualize treatment and properly assess outcomes. It should also be noted that direct massage of the tumor, lymph nodes connected to the tumor and sites of bone metastases, radiotherapy and thrombus occurrence should be avoided; moreover, patients with low platelet counts should avoid massage treatment. Recommendation 9 sums up the position of the consensus committee regarding complementary therapies, and expressly notes that the attending physician should be informed before any therapy is initiated.

#### Benefits of combined intervention

As CRF is often multicausal in nature, a combination of non-pharmacological interventions may have a better effect on reducing CRF (32,71). For example, recent meta-analyses have shown that CBT combined with physical activity (32) and multimodal therapy (71) offer advantages in alleviating CRF, while individual studies also indicate that benefits can be gained by combining psychosocial interventions with exercise or by combining different non-pharmacological interventions such as patient education, exercise, CBT and sleep hygiene (49,56,57,72).

### Pharmacological treatment

Evidence regarding the effectiveness of pharmacological therapies in CRF is quite limited, and there are very few medications that are indicated in the treatment of CRF. The consensus committee has therefore included only medications for which RCT evidence is available.

#### Methylphenidate

Methylphenidate, a psychostimulant approved for treatment of attention deficit hyperactivity disorder, has been suggested for use in CRF. However, existing trials offer only limited evidence, and larger trials are needed to validate the use of methylphenidate in the treatment of CRF (73–76). Meta-analyses of small-sized trials have demonstrated that a subset of patients with more severe fatigue and/or with more advanced disease did experience some fatigue improvement with methylphenidate (75,76). The consensus committee therefore recommends that methylphenidate might be useful for the relief of CRF, but also notes that RCTs involving the use of methylphenidate at doses lower than 10–30 mg/day or at a duration shorter than 6 weeks showed that such treatment was not effective for the relief of CRF (77,78), and therefore, appropriate dosage and duration is needed. The consensus committee further highlights the risk of adverse reactions and abuse with methylphenidate (79,80) and urges careful consideration of the characteristics of each individual patient prior to initiating treatment. Treatment- or disease-specific morbidities should be characterized or excluded before using methylphenidate. It should also be noted that methylphenidate has no officially approved indication for the treatment of CRF in Taiwan, and it should not be used as the sole treatment for CRF under any circumstance. Recommendation 10 emphasizes that the use of methylphenidate should be carefully considered prior to initiating treatment.

#### Methylprednisolone and dexamethasone

Steroids are believed to be able to reduce CRF by alleviating inflammatory conditions. Evidence from RCTs show that methylprednisolone can improve fatigue, appetite, and QoL in cancer patients (81,82), while dexamethasone was shown to better improve fatigue over placebo (83). However, the consensus committee also noted the side effects associated with long-term use of steroids, and therefore recommends such treatment is only for patients with terminal disease, combined with fatigue, anorexia or pain caused by brain or bone metastasis, in accordance with the latest NCCN CRF guidelines (14).

#### *Astragalus membranaceus* polysaccharides injection

*Astragalus membranaceus* polysaccharides, extracted, isolated and purified from *A. membranaceus*, with a molecular weight of 20–60 kiloDaltons (kDa) (84), have been shown to promote the release of growth factors for granule leukocytes and hematopoietic stem cells (84), reduce inflammatory factors such as TNF-α, IL-6, IL-10, MCP-1, IFN-γ and IL-1β (85,86), increase the M1/M2 macrophage polarization ratio (86) and upregulate the expression of *PTPN11* and *NFKB2* genes to affect the proliferation of blood cells and immune cells (87). In RCTs, *A. membranaceus* polysaccharides injection significantly improved fatigue, appetite, QoL, physical fitness, nausea, vomiting and pain in cancer patients (84,88–90). In a Phase II/III pivot trial, cancer patients with moderate to severe fatigue (as defined by fatigue score ≥ 4 on the Brief Fatigue Inventory-Taiwan) who received 500 mg daily of *A. membranaceus* polysaccharides injection demonstrated significant improvement in fatigue scores as compared with patients who received placebo, while the incidence and severity of adverse reactions were not significantly increased as compared with placebo (84). Based on the results of this study, *A. membranaceus* polysaccharides injection was approved by the Taiwan Food and Drug Administration in 2015 for the treatment of moderate to severe fatigue symptoms induced by disease progression in terminal cancer patients. A subsequent double-blind, multicenter Phase IV RCT found that *A. membranaceus* polysaccharides injection improved fatigue scores by 10% or more in 65% of patients after just one cycle of treatment (90). The consensus committee therefore elected to include this treatment in Recommendation 12, but also noted that further clinical studies in other populations may be warranted for a broader indication.

### Traditional herbal medicine

Several large studies have investigated the efficacy of ginsengs (including Asian ginseng, *Panax ginseng*, and American ginseng*, Panax quinquefolius*) in the management of CRF, and the results indicate that the dosage, duration, type and preparation process of ginsengs may influence their efficacy against CRF. For example, one RCT showed that 400 mg of Asian ginseng standardized powder given twice daily for 28 days could only improve fatigue significantly in male cancer patients or those with greater fatigue or depression (91), while another RCT showed that 2000 mg of Korean red ginseng (steamed Asian ginseng) powder given daily over 16 weeks significantly improved fatigue and QoL (92). Studies conducted with American ginseng showed that daily doses of 1000 mg or 2000 mg ginseng powder given for 8 weeks were more effective in reducing fatigue than a daily dose of 750 mg or placebo (93,94). It has been suggested that this is because standardized American ginseng products contain at least 3% of ginsenosides, with fewer additives (95). The consensus committee agreed that ginsengs may be effective in reducing fatigue. However, considering the variations in the manufacturing process among various commercially available ginseng products, it was stated in Recommendation 13 that patients should consult their healthcare teams prior to initiating treatment with ginsengs.

## Discussion

This consensus comprises 13 recommendations on the screening, assessment and management of CRF based on the best available evidence. The consensus aims to standardize clinical practices for CRF in Taiwan, and CME activities for this consensus have been continuously conducted across Taiwan since its formulation. Currently, courses on this consensus are included in the core curriculum for early palliative cancer care at both TSCPM and TONS. Periodic surveys are also being conducted to evaluate awareness and implementation of the consensus in clinical practice, and the results of these surveys, as well as new evidence pertaining to the assessment and management of CRF, will be used to regularly update and improve this consensus. In addition, since 2018, implementation of this consensus has been included as a bonus item in accreditation reviews of quality cancer care at cancer centers across Taiwan. Since the publication of the Chinese version of this consensus in 2017, the large-scale CME activities, CRF surveys aimed at both patients and healthcare personnel and policy support from regulatory authorities in hospital review and accreditation have greatly increased awareness and concern regarding CRF management and will hopefully achieve a defining impact on the enhancement of CRF care over the long term. It is hoped that through such measures, issues related to the under-detection and under-treatment of CRF in Taiwanese cancer patients can be effectively resolved. Another survey of cancer patients across Taiwan regarding CRF prevalence, treatment and outcomes is currently being planned to assess the clinical impact of the consensus over the past 5 years.

This consensus is unique in that the evidence for non-pharmacological and pharmacological treatments has been extensively reviewed, and recommendations regarding a broad range of non-pharmacological treatments, including complementary therapies, have also been included, as questions regarding such therapies are frequently encountered in clinical practice in Taiwan. The consensus also presents a recommendation and accompanying evidence regarding a novel treatment, *A. membranaceus* polysaccharides injection, which has been approved in Taiwan since 2015 for the treatment of moderate to severe fatigue symptoms induced by disease progression in advanced cancer patients.

There are some limitations to the current consensus. Firstly, there is only limited evidence available to support the efficacy of specific non-pharmacological or pharmacological interventions for the improvement of CRF, and more large-scale studies are needed to provide a stronger evidence base for any clinical recommendations. Secondly, the causes of fatigue may differ from patient to patient, and individual assessments are still quite important in informing the most effective course of treatment. This consensus aims to increase awareness among clinicians, nurses and other healthcare personnel regarding the prevalence and impact of CRF, but further evidence will be needed to establish a more detailed management algorithm that can be applied to specific patient groups. Thirdly, it is hoped that as this consensus is effectively implemented in the clinical setting, there will be more clinical experience and observational studies to inform best practices in the assessment and management of CRF, and this will allow the consensus to become more effective. Currently, the consensus relies on internationally recognized guidelines such as the NCCN guidelines to help provide guidance on clinical practices where the evidence may be lacking, but as more local knowledge is built up, it may be possible to develop and implement recommendations that are more applicable and effective in local populations.

## Conclusions

CRF is a common but under-managed problem that impairs cancer patients physically, mentally and socially. It may also disturb the course of anticancer therapies. Therefore, clinicians, nursing staff and other healthcare personnel should learn to recognize and manage CRF effectively, and it is hoped that the development of this consensus can serve to increase awareness and facilitate the implementation of best practices in CRF screening, assessment and treatment for the benefit of patients.
